# Supplementary material for: Empirical methods for controlling false positives and estimating confidence in ChIP-Seq peaks
Source: BMC Bioinformatics. 2008 Dec 5;9:523. doi: 10.1186/1471-2105-9-523 (PMC2628906; doi:10.1186/1471-2105-9-523)
Supplement: Additional File 1 — A variety of html documents from the USeq web site detailing the available applications, their best usage, output file type descriptions, command line menus, etc. [file 1471-2105-9-523-S1.zip › USeqUserGuides/JavaDoc.zip!/JavaDoc/trans/graphics/GenomicRegion.html]

GenomicRegion


|  |  |  |
| --- | --- | --- |
| **Overview** \| **Package** \| **Class** \| **Use** \| **Tree** \| **Deprecated** \| **Index** \| **Help** | |  |
| **PREV CLASS**   **NEXT CLASS** | **FRAMES**    **NO FRAMES**     **All Classes** |  |
| SUMMARY: NESTED \| FIELD \| CONSTR \| METHOD | DETAIL: FIELD \| CONSTR \| METHOD |  |


---


## trans.graphics Class GenomicRegion

```
java.lang.Object
  trans.graphics.GenomicRegion
```

---

``` public class GenomicRegion extends java.lang.Object ```

---

**Constructor Summary**

`GenomicRegion(java.lang.String line,
int rank)`


| **Method Summary** | |
| --- | --- |
| `static int` | `findBiggestGenomicRegion(GenomicRegion[] gr)`             Returns the length of the biggest GenomicRegion |
| `java.lang.String` | `getChromosome()` |
| `GenomicRegionGlyph` | `getGlyph()` |
| `java.util.ArrayList` | `getIntersectingRegions()` |
| `java.lang.String` | `getNotes()` |
| `int` | `getRank()` |
| `double` | `getScore()` |
| `int` | `getStart()` |
| `int` | `getStop()` |
| `boolean` | `overlap(GenomicRegion other, int maxGap)`             checks to see if regions overlap by the minimum maxGap, can set maxGap negative to require an overlap. |
| `void` | `setGlyph(GenomicRegionGlyph glyph)` |
| `void` | `setRank(int rank)` |
| `java.lang.String` | `toString()`             Returns tab delimited rank+1, chrom, st, stp, scr, notes. |

**Methods inherited from class java.lang.Object**

`equals, getClass, hashCode, notify, notifyAll, wait, wait, wait`

**Constructor Detail**

### GenomicRegion

```
public GenomicRegion(java.lang.String line,
                     int rank)
```


**Method Detail**

### toString

```
public java.lang.String toString()
```

:   Returns tab delimited rank+1, chrom, st, stp, scr, notes.

    :   **Overrides:**: `toString` in class `java.lang.Object`

---


### overlap

```
public boolean overlap(GenomicRegion other,
                       int maxGap)
```

:   checks to see if regions overlap by the minimum maxGap, can set maxGap negative to require an overlap.

---


### findBiggestGenomicRegion

```
public static int findBiggestGenomicRegion(GenomicRegion[] gr)
```

:   Returns the length of the biggest GenomicRegion

---


### getChromosome

```
public java.lang.String getChromosome()
```

---


### getIntersectingRegions

```
public java.util.ArrayList getIntersectingRegions()
```

---


### getNotes

```
public java.lang.String getNotes()
```

---


### getScore

```
public double getScore()
```

---


### getStart

```
public int getStart()
```

---


### getStop

```
public int getStop()
```

---


### getGlyph

```
public GenomicRegionGlyph getGlyph()
```

---


### setGlyph

```
public void setGlyph(GenomicRegionGlyph glyph)
```

---


### getRank

```
public int getRank()
```

---


### setRank

```
public void setRank(int rank)
```


---


|  |  |  |
| --- | --- | --- |
| **Overview** \| **Package** \| **Class** \| **Use** \| **Tree** \| **Deprecated** \| **Index** \| **Help** | |  |
| **PREV CLASS**   **NEXT CLASS** | **FRAMES**    **NO FRAMES**     **All Classes** |  |
| SUMMARY: NESTED \| FIELD \| CONSTR \| METHOD | DETAIL: FIELD \| CONSTR \| METHOD |  |


---
